# 

**Published:** 1893-10-21

**Authors:** 


					^j[ "ll " J J Jli/,
WITH EXTRA NURSING SUPPLEMENT.
Per Annum, 8s. 6d., post free.
Monthly Farts, 6d.; post free, 8d.
Ditto per Annum, 8s., post free.
No. 369. Vol. XV. Satukday,
October 21st, 1893.
[Twopence.

				

